# Bioactive Volatiles from an Endophytic *Daldinia cf*. *concentrica* Isolate Affect the Viability of the Plant Parasitic Nematode *Meloidogyne javanica*

**DOI:** 10.1371/journal.pone.0168437

**Published:** 2016-12-20

**Authors:** Orna Liarzi, Patricia Bucki, Sigal Braun Miyara, David Ezra

**Affiliations:** 1 Department of Plant Pathology and Weed Research, ARO - the Volcani Center, Rishon LeZion, Israel; 2 Department of Entomology and the Nematology and Chemistry units, ARO - the Volcani Center, Rishon LeZion, Israel; James Hutton Institute, UNITED KINGDOM

## Abstract

Plant-parasitic nematodes form one of the largest sources of biotic stress imposed on plants, and are very difficult to control; among them are the obligate parasites, the sedentary root-knot nematodes (RKNs)–*Meloidogyne* spp.–which are extremely polyphagous and exploit a very wide range of hosts. Endophytic fungi are organisms that spend most of their life cycle within plant tissue without causing visible damage to the host plant. Many endophytes secrete specialized metabolites and/or emit volatile organic compounds (VOCs) that exhibit biological activity. Recently, we demonstrated that the endophytic fungus *Daldinia cf*. *concentrica* secrets biologically active VOCs. Here we examined the ability of the fungus and its VOCs to control the RKN *M*. *javanica* both *in vitro* and greenhouse experiments. The *D*. *cf*. *concentrica* VOCs showed bionematicidal activity against the second-stage juveniles (J2s) of *M*. *javanica*. We found that exposure of J2s to fungal volatiles caused 67% reduction in viability, and that application of a synthetic volatile mixture (SVM), comprising 3-methyl-1-butanol, (±)-2-methyl-1-butanol, 4-heptanone, and isoamyl acetate, in volumetric ratio of 1:1:2:1 further reduced J2s viability by 99%. We demonstrated that, although each of the four VOCs significantly reduced the viability of J2s relative to the control, only 4-heptanone elicited the same effect as the whole mixture, with nematicidal activity of 90% reduction in viability of the J2s. Study of the effect of the SVM on egg hatching demonstrated that it decreased eggs hatching by 87%. Finally, application of the SVM to soil inoculated with *M*. *javanica* eggs or J2s prior to planting susceptible tomato plants resulted in a significantly reduced galling index and fewer eggs produced on each root system, with no effect on root weight. Thus, *D*. *cf*. *concentrica* and/or SVM based on fungal VOCs may be considered as a novel alternative approach to controlling the RKN *M*. *javanica*.

## Introduction

Among the most devastating plant pathogenic nematodes are the sedentary root-knot nematodes (RKNs)–*Meloidogyne* spp.–which are obligate biotrophs [1]. These parasites establish sophisticated communication with selected host cells that is mediated by nematode-secreted effectors, and by which they alter host-cell development and gene expression. As a result, the host forms large multinucleate cells, known as giant cells, which constitute the sole source of nutrition for the developing nematode [2–5]. Concurrent above-ground symptoms include stunted growth and wilting, caused by restricted water transport through the disrupted plant vascular elements and by alterations in nutrient partitioning [6]. Management strategies, including crop rotation, are vitiated by the broad host range of *Meloidogyne* spp. Chemical means of control face increasingly restrictive regulations because most nematicides are non-specific, notoriously toxic, and pose a potential threat to the soil ecosystem, ground water, and human health [7, 8]. Alternative means to control *M*. *javanica* utilized fungi [9–12], bacteria [13–20], and organic amendments [21].

Endophytes are microorganisms that spend part of their life cycle within plant tissues without causing any visible damage.

Accumulating evidence shows that endophytes benefit plants by promoting plant growth and thereby increasing crop yields, and confer tolerance to both biotic and abiotic stresses [22–26]. The former stresses are imposed by interspecific competition, invertebrate pests, herbivory by mammals, and diseases caused by phytopathogens; the latter are due to heavy-metal pollution, drought, salinity, and unfavorable temperatures.

Many endophytes have been found to secrete specialized metabolites and complex glycoproteins [27–31], and some endophytic fungi emit volatile organic compounds (VOCs) [32–37] that may be biologically active, for example, as a biofumigant for controlling postharvest disease [38]. *Muscodor albus* is an endophytic fungus that produces and emits VOCs that are capable of killing a broad range of fungi and bacteria that are pathogenic against plants and humans [29, 39, 40]. *M*. *albus* also exhibits nematistatic and nematicidal properties against plant-parasitic nematodes [41, 42]. Nematicidal VOCs were observed in the model yeast *Saccharomyces cerevisiae*: exposure of second-stage juveniles of *M*. *javanica* to a synthetic mixture of the yeast VOCs resulted in nematode mortality [43]. Similarly, VOCs produced by *Fusarium oxysporum* isolated from rhizospheres of coffee plants were nematicidal against *M*. *incognita* nematodes [44].

The fungus *Daldinia concentrica* belongs to the Xylariaceae family, which typically is found on decaying wood [45]. *Daldinia* species can also be found as endophytes in orchids (*D*. *eschscholtzii*) [46] and in insects (*D*. *hawksworthii*) [47]. Recently, our laboratory obtained an endophytic *D*. *cf*. *concentrica* isolate from an olive tree (*Olea europaea* L.) growing in Israel. This isolate was found to emit VOCs that exhibited antimicrobial activity against various plant pathogenic fungi; it also exhibited protective effects on stored dried fruits and peanuts [48]. A SVM, based on identified VOCs emitted by *D*. *cf*. *concentrica*, exhibited activity similar to or more potent than that of the fungus itself [48]. In the present study we aimed to exploit all the accumulated findings and to study the potential of *D*. *cf*. *concentrica* and/or a SVM of its VOCs for management of *M*. *javanica*.

## Materials and Methods

### Fungal culture

The *D*. *cf*. *concentrica* isolate was obtained as an endophyte from a branch of an olive tree (*Olea* *europaea* L.) located in the Ha'Ela Valley in the Judean Hills in Israel (N 31.681915, E 34.988792). Wood fragments were surface-sterilized by immersion in ethanol for 10 s, followed by flaming. Then, small pieces were cut and placed on potato dextrose agar (PDA) (Acumedia, Lansing, Michigan, USA) amended with tetracycline at 12 μg/mL (Sigma, Rehovot, Israel), and incubated at 25°C. After 5 days, isolated fungal hyphal tips emerged from the plant material onto the PDA. Then, mycelial fragments were removed with a sterile scalpel and transferred to a new PDA-tetracycline plate. A single spore colony was used throughout this study. Isolate *D*. *cf*. *concentrica* was routinely maintained on PDA-tetracycline plates, and incubated at 25°C. Fresh fungal mycelium was transferred to a new plate every 2 weeks. Sequences of internal transcribed spacer and partial actin gene were submitted to GenBank and deposited as accession numbers EU201138 and FJ269018, respectively [48]. Isolate *D*. *cf*. *concentrica* was deposited in the restricted collection of the CBS culture bank as culture CBS123047.

### Nematode preparation

*Meloidogyne javanica* was propagated on greenhouse-grown nematode-susceptible tomato *Lycopersicon esculentum* cv. 'Avigail 870' (Hazera, Shikmim, Israel), and nematode eggs were bulk-extracted from the roots with 0.05% (v/v) sodium hypochlorite (NaOCl) followed by sucrose flotation and successive sievings through nylon filters of 300, 60, and 30 μm mesh sizes (AD Sinun Technologies, Petach Tikvah, Israel) [49]. For hatching J2, extracted eggs were placed on 30-μm sieves (AD Sinun Technologies) in 0.01 M MES (2-(N-morpholino) ethanesulfonic acid hydrate) (Sigma) pH 6.5 buffer under aseptic conditions in darkness for 3 days, and hatching J2s were collected for further experiments. For soil-inoculation tests, heavily infected, susceptible tomato roots cv. 'Avigail 870' (Hazera) were processed in a Waring commercial blender (Waring, Torrington, CT, USA) for 2 min at high speed. A small sample of the blended roots was taken for evaluation of eggs concentration by means of 0.05% (v/v) sodium hypochlorite (NaOCl) extraction, as described above. The blended roots were used for soil inoculation in the subsequent experiments.

### *In vitro* experiments setup with *D*. *cf*. *concentrica* culture

For *in vitro* studies of the bionematicidal activity of *D*. *cf*. *concentrica* toward *M*. *javanica* J2s, pure culture plates of the fungus were prepared as follows: a plug of *D*. *cf*. *concentrica* was transferred to a 50-mm Petri plate containing 5 mL of potato dextrose broth (PDB; Acumedia, Lansing, Michigan, USA), and was allowed to grow for 4–5 days for use in the subsequent experiments. *M*. *javanica* eggs or J2 were obtained as described above. *In vitro* tests were performed in duplicate for each treatment, in three independent experiments. The experiments took place in sealed 1-L boxes, each of which contained one to three uncovered culture plates of *D*. *cf*. *concentrica*, as well as five small vials measuring 12 × 35 mm (S Murray & Co, Surrey, England), each containing 300 *M*. *javanica* J2s in 0.5 mL of 0.01M MES. In order to maintain a humid environment, each sealed box was equipped with a 50-mm Petri plate containing 5 mL of sterile double-distilled H_2_O. A 5-mL PDA plate cultured with a fresh plug of *Aspergillus niger* also was placed in each sealed box. Inhibition of *A*. *niger* by *D*. *cf*. *concentrica* was reported previously [48], and was used as a positive internal control in our present system. Possible effect of *A*. *niger* on J2s viability was examined by comparing the number of viable J2s after exposure to *A*. *niger* and to unexposed ones under the same conditions. The sealed boxes containing the above-described biological agents were incubated for 2 days, after which J2s were carefully washed through 30 μm sieve, later allowed to actively pass (within 3 h) the sieve and counted. All incubations were performed in the dark at 25 ± 1°C.

### *In vitro* experiments using SVM

All compounds used were of the highest available purity and were purchased from Sigma (Rehovot, Israel). The SVM contained 3-methyl-1-butanol (3-Methylbutan-1-ol), (±)-2-methyl-1-butanol (2-Methylbutan-1-ol), 4-heptanone (Heptan-4-one), and isoamyl acetate (3-Methylbutyl acetate) in volumetric ratio of 1:1:2:1. Each sealed 1-L box was arranged as described above, except that the *D*. *cf*. *concentrica* culture plates were replaced with one single (12 × 35)-mm vial (S Murray & Co) containing 1 mL/L (V/V) of the SVM [3-methyl-1-butanol (0.2 mL, 1.84 mmole), (±)-2-methyl-1-butanol (0.2 mL, 1.86 mmole), 4-heptanone (0.4 mL, 2.86 mmole), and isoamyl acetate (0.2 mL, 1.35 mmole)], and no *A*. *niger* culture plate was used. The boxes, which were duplicated for each treatment in three independent experiments, were sealed and incubated as described above, after which viable J2s were counted. The nematicidal activity of each individual compound was determined as described for the SVM, except that each duplicated box in each of three independent experiments contained one vial. The vial was loaded with the chemicals, in the same proportions as in the SVM: 3-methyl-1-butanol (0.2 mL, 1.84 mmole), (±)-2-methyl-1-butanol (0.2 mL, 1.86 mmole), 4-heptanone (0.4 mL, 2.86 mmole), and isoamyl acetate (0.2 mL, 1.35 mmole). For determination of the nematicidal activity of 4-heptanone, the following treatments were used, each involving 0.4 mL, 2.86 mmole of 4-heptanone, duplicated in each of three independent experiments: a) incubation of nematodes for 24 h in the presence of 4-heptanone; b) incubation of nematodes for 24 h in the absence of 4-heptanone; c) incubation of nematodes for 24 h in the presence of 4-heptanone followed by their transfer to new boxes without the compound for an additional 24 h, for recovery; d) incubation of nematodes for 48 h in the presence of 4-heptanone; and e) incubation of nematodes for 48 h without the compound. The boxes were prepared with the nematodes and water as described above. All incubations were performed in darkness at 25 ± 1°C.

A suspension of *M javanica* eggs, extracted with NaOCl and then concentrated, was used to study the effects of the fungus and SVM on hatching. The experimental setup comprised sealed 1-L boxes as follows. Control boxes contained 5 mL of sterile double-distilled H_2_O as a source of humidity, five (12 × 35)-mm vials (S Murray & Co), each containing 800 *M*. *javanica* eggs in 0.5 mL of 0.01M MES, and one uncovered 50-mm Petri plate containing 5 mL of PDA and a fresh plug of *A*. *niger*. The boxes containing *D*. *cf*. *concentrica* and SVM were prepared similarly to the control boxes, except that three uncovered culture plates of *D*. *cf*. *concentrica*–pre-grown for 5 days—were added to the former, and one 50-mm Petri plate containing 125 mg of perlite particles loaded with 0.25 mL/L (V/V) of the SVM [3-methyl-1-butanol (0.05 mL, 0.46 mmole), (±)-2-methyl-1-butanol (0.05 mL, 0.46 mmole), 4-heptanone (0.1 mL, 0.72 mmole), and isoamyl acetate (0.05 mL, 0.34 mmole)]was added to the latter. The duplicated boxes for each treatment in each of three independent experiments were incubated for 2 days. Then, the *M*. *javanica* eggs were collected from the vials, loaded onto 30-μm filters (AD Sinun Technologies), and inserted into 15-mL Falcon centrifuge tubes, each containing 900 μL of 0.01M MES buffer, in order to remove larvae that had hatched before completion of exposure of the eggs to the volatiles. After 3 h of incubation the filters were transferred to new 15-mL Falcon tubes containing fresh MES buffer as above, and incubated for two additional days for hatching. The viable J2s that hatched from eggs that had been exposed to the volatiles for 2 days were counted with a hemocytometer as described below, and the percentage of egg hatching was determined by calculating the ratio between the number of viable J2s in each vail and the number of eggs inserted (800 eggs). All incubations were performed in the dark at 25 ± 1°C.

### Nematodes J2s viability

To evaluate second-stage juveniles viability following exposure to fungal culture plates or the SVM, we conducted a straightforward small scale nematode extraction method which is based on the traditional Baermann funnel method [50]. Briefly, all nematodes suspensions were loaded onto 30-μm (AD Sinun Technologies) filters (1.0–1.5 cm height, 1.1–2.0 cm^3^ internal area), and nematodes were washed twice with 300 μL of 0.01M MES buffer to remove any volatiles residues. Then, filters holding nematodes were placed into 15-mL Falcon centrifuge tubes, each containing 900 μL of 0.01M MES buffer. These filters allow active passage of only living J2s while paralyzed or dead nematodes remain on top of the filter. The whole apparatus including the filter holding the nematodes and the Falcon tubes were placed in the dark for 3 h at 25 ± 1°C. Then, the filters were removed and the viable nematodes, which were able to actively cross the filter toward the 0.01M MES buffer, were collected from the Falcon tube and counted using a Nematode Counting Slide (Chalex LLC, Portland, OR, USA) and a Wilovert Standard inverted microscope (Helmut Hund GmbH, Wetzlar, Germany).

### Soil experimental setup

For each biological repetition, a stock of SVM (0.4 mL) was prepared by mixing 3-methyl-1-butanol (0.08 mL, 0.73 mmole), (±)-2-methyl-1-butanol (0.08 mL, 0.74 mmole), 4-heptanone (0.16 mL, 1.14 mmole), and isoamyl acetate (0.08 mL, 0.54 mmole). For each technical repetition, 5, 10, 15, 20, or 30 μL of the stock SVM was loaded on perlite particles (1:1 ratio between perlite particles and mixture), poured into a 50-mL plastic cup containing 60 g of loam soil of 10% humidity (Givat Ada, Israel), and shaken thoroughly till complete homogenization of the perlite in the soil. In control treatment, 30 mg of perlite particles were used with no SVM. The resultant concentrations of the SVM in the soil were 0, 0.08, 0.17, 0.25, 0.33, and 0.5 μL/gr soil. Then, 500 *M*. *javanica* J2s in 0.5 mL of 0.01M MES were placed in a small pit in the soil surface. The cups (five cups per concentration in each of two independent experiments) were sealed and incubated for 2 days in the dark at 25 ± 1°C. Viable J2 nematodes were determined according to the Baermann funnel method [50].

### Greenhouse experimental setup

Twenty pots were loaded with 800 gr of loam soil at 10% humidity and supplemented with Osmocote^®^ slow-release fertilizer (Scotts, Bella Vista, NSW, Australia). Perlite particles (400 mg), with or without SVM (0.4 mL) [3-methyl-1-butanol (0.08 mL, 0.73 mmole), (±)-2-methyl-1-butanol (0.08 mL, 0.74 mmole), 4-heptanone (0.16 mL, 1.14 mmole), and isoamyl acetate (0.08 mL, 0.54 mmole)], were added to each pot and shaken till homogenization. Half of the pots (10 pots) were each inoculated with 4,000 *M*. *javanica* larvae (J2) in 5 mL of 0.01M MES, whereas the other ten pots were each inoculated with 5 mL of a suspension of nematode-infected roots (NIR), equivalent to ~4,000 *M*. *javanica* eggs. We used specific weight of infected roots containing females and egg masses equivalent to 4000 eggs per pot. Five pots from each inoculation method contained the SVM, and the other 5 pots contained only perlite particles without the mixture as a control. All pots were sealed and incubated for 3 days in the dark at 25 ± 2°C. Then the covers were removed and 4-week-old susceptible tomato seedlings, cv. Avigail 870 (Hazera) were planted. The seedlings were grown for 7 weeks at 25 ± 2°C under 16/8 h day/night conditions, irrigated with water at 20 mL/day, after which parasitism development was evaluated. The greenhouse experiment was repeated as above, except that the soil was inoculated with nematodes—either in the form of J2s or as a suspension of NIR– 3 days before addition of the SVM. Then, the nematode-inoculated soil and the volatile mixture were mixed, sealed and incubated for an additional 3 days in the dark at 25 ± 2°C before tomatoes were planted. The seedlings were grown for 8 weeks as described above.

At the end of the experiments the plants, along with their root systems, were harvested from their pots. For evaluating parasitism development on roots, soil debris was carefully removed from washed root systems, incidences of root galling were visually evaluated, and roots were weighed. To evaluate nematode reproduction on root systems, *M*. *javanica* eggs were extracted from each root as described [51] and counted, and the numbers of eggs per gram of root were calculated.

### Statistical analysis

Data were analyzed with the JMP 10 software package (SAS Inc., Cary, NC, USA). Mean numbers of viable *M*. *javanica* J2s under various conditions were subjected to one-way analysis of variance (ANOVA), followed by the Tukey-Kramer multiple comparison test, with significance set at *P* < 0.05. Galling indexes were subjected to nonparametric comparison using Wilcoxon method.

### Direct visualization of nematodes

Either 1,000 or 3,000 *M*. *javanica* J2s in 0.5 mL of 0.01M MES were transferred into (12 × 35)-mm glass vials within humid, sealed 1-L boxes and exposed to either: three 50-mm culture plates of pre-grown *D*. *cf*. *concentrica*; 0.25 mL/L (V/V) [3-methyl-1-butanol (0.05 mL, 0.46 mmole), (±)-2-methyl-1-butanol (0.05 mL, 0.46 mmole), 4-heptanone (0.1 mL, 0.72 mmole), and isoamyl acetate (0.05 mL, 0.34 mmole)] of SVM preloaded onto 125 mg of perlite particles; or 4-heptanone (0.05 mL, 0.36 mmole) preloaded onto 25 mg of perlite particles. Vials in a sealed box without any treatment served as controls. After 2 days of incubation in the dark at 25 ± 1°C duplicated samples of 1,000 or 3,000 J2s were combined and centrifuged (Eppendorf, Hamburg, Germany) for 3 min at 210 *g*. A 50-μL drop from the bottom of the tube was used for direct visualization under a Leica MZFLIII stereomicroscope (Leitz, Wetzlar, Germany) with the aid of a Nikon DS-Fi1 digital camera and NIS-Elements software (Nikon, Tokyo, Japan).

## Results

### *Effects on* larval viability

**Fig 1:**
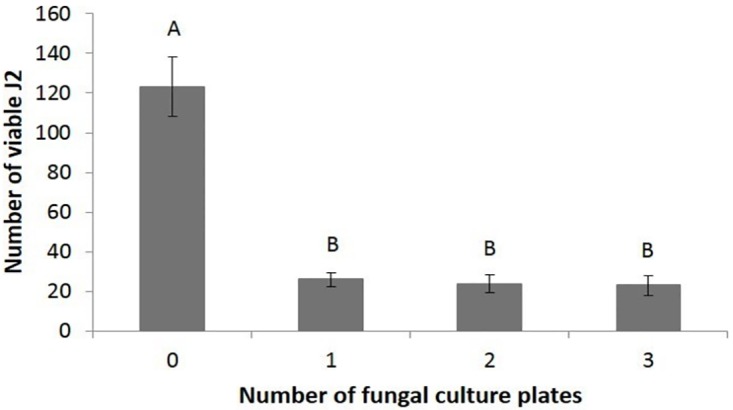
Effect of *D*. *cf*. *concentrica* culture plate on *M*. *javanica* J2 viability. J2s were exposed to 0–3 culture plates of *D*. *cf*. *concentrica* for 48 h. The numbers on the x-axis represent the numbers of Petri plates (50 mm in diameter, containing 5 mL of growth medium, and the fungal culture) in each 1-L sealed box. The number 0 indicates control treatment in which the nematodes were not exposed to the fungal culture plate. The viable J2s were separated using 30 μm sieves, and the numbers on the y-axis represent the numbers (mean ± SE) of viable J2s counted following the incubation. There were 10 repetitions, each using 300 J2s. The results were subjected to analysis of variance followed by the Tukey-Kramer multiple comparison test; different letters above the bars indicate a significant difference between samples at *P ≤* 0.05. The experiment was independently repeated three times, each time with similar results.

The potential biocontrol activity of *D*. *cf*. *concentrica* against *M*. *javanica* was studied on J2-stage larvae in sealed boxes that contained one, two, or three uncovered fungal culture plates. In this experimental setup there was no contact between the J2s and the fungus, therefore any effect of the fungus on larval viability could be only via its emitted volatile compounds. The presence of *A*. *niger* in the experimental setup, as a positive internal control for the activity of *D*. *cf*. *concentrica* [48], had no significant effect on J2s viability: 112.6±10.2 and 96.9±9.5 (mean±SE) for control and *A*. *niger* exposed J2s, respectively, with no significant differences (P-Value = 0.27) between treatments according to one way ANOVA. As shown in Fig 1, *D*. *cf*. *concentrica* significantly reduced the number of viable *M*. *javanica* J2s, already in the presence of one fungus culture plate. The mean reduction in viability of *M*. *javanica* J2s in the presence of *D*. *cf*. *concentrica* was 69.1 ± 15.1, 66.4 ± 15.7, and 66.3 ± 14% following exposure to one, two, or three culture plates, respectively. It should be noted that although *D*. *cf*. *concentrica* significantly affected *M*. *javanica* J2s, it exhibited only weak nematicidal behavior (data not shown).

### Improved biological activity of SVM based on *D*. *cf*. *concentrica* emitted VOCs

**Fig 2:**
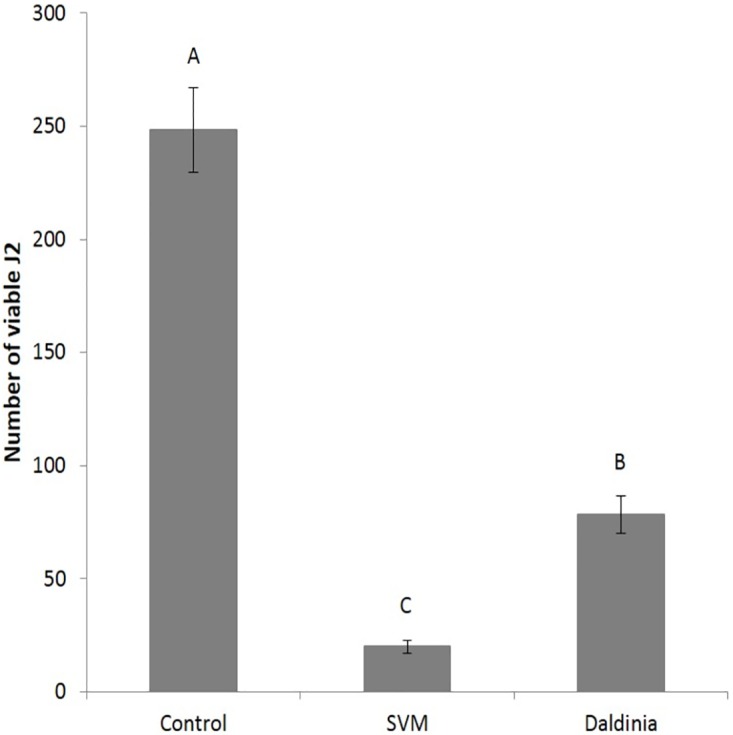
Effect on *M*. *javanica* J2 viability of a SVM compared with that of *D*. *cf*. *concentrica* culture. J2s were exposed for 48 h either to the SVM at 1 mL/L (V/V) [3-methyl-1-butanol (0.2 mL, 1.84 mmole), (±)-2-methyl-1-butanol (0.2 mL, 1.86 mmole), 4-heptanone (0.4 mL, 2.86 mmole), and isoamyl acetate (0.2 mL, 1.35 mmole)] or to three 50-mm-diameter fungal culture plates, each with 5 mL of growth medium, and then the viable J2s were separated using 30 μm sieves. The numbers on the y-axis represent the numbers (mean ± SE) of viable J2s counted following the incubation. Each treatment was composed of 10 technical repetitions using 300 J2s in each repetition. The results were subjected to analysis of variance followed by the Tukey-Kramer multiple comparison test; different letters above the bars indicate a significant difference between samples at *P ≤* 0.05. The experiment was independently repeated three times, each time with similar results.

As described elsewhere [48], *D*. *cf*. *concentrica* emits biologically active VOCs. Therefore we examined whether the biological activity of *D*. *cf*. *concentrica* could be mimicked by a SVM comprising the following emitted fungal VOCs in the same 1:1:2:1 ratio previously demonstrated to be bioactive [48]: 3-methyl-1-butanol, (±)-2-methyl-1-butanol, 4-heptanone, and isoamyl acetate. Fig 2 shows that exposure of the J2s to the mixture elicited a sharp reduction in their viability: 88.1 ± 4.0 and 65.1 ± 3.3% reduction in J2s viability could be observed for juveniles treated by the SVM or *D*. *cf*. *concentrica* culture plates, respectively. This result suggests that the effect of the SVM on larval viability was stronger than the biological activity of *D*. *cf*. *concentrica* VOCs.

**Fig 3:**
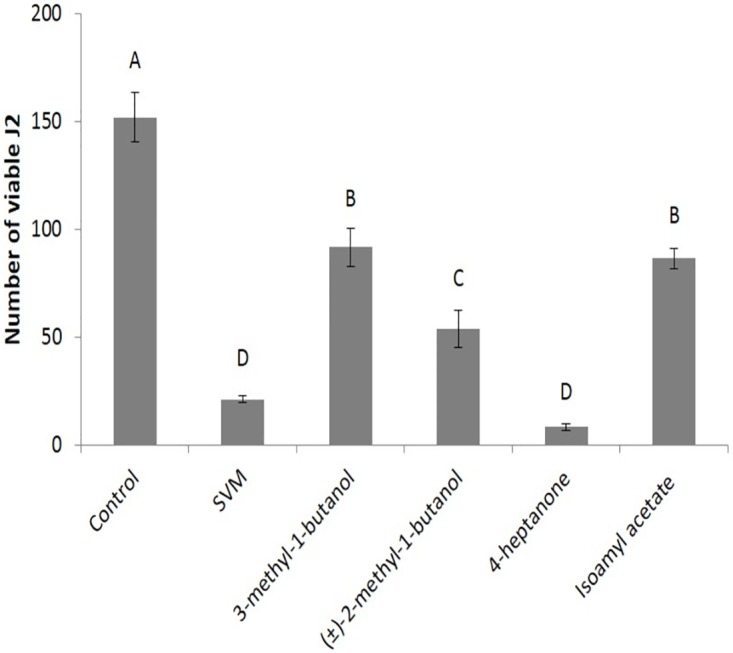
Effects of individual compounds on *M*. *javanica* J2 viability. J2s were separately exposed for 48 h to 3-methyl-1-butanol (0.2 mL, 1.84 mmole), (±)-2-methyl-1-butanol (0.2 mL, 1.86 mmole), 4-heptanone (0.4 mL, 2.86 mmole), isoamyl acetate (0.2 mL, 1.35 mmole), or to 1 mL combination thereof (SVM), before viable J2s were separated using 30 μm sieves. The numbers on the y-axis represent the numbers (mean ± SE) of viable J2s counted following the incubation. There were 7–10 repetitions, each using 300 J2s. The results were subjected to analysis of variance followed by the Tukey-Kramer multiple comparison test; different letters above the bars indicate a significant difference between samples at *P ≤* 0.05. The experiment was independently repeated three times, each time with similar results.

We examined the viability of *M*. *javanica* J2s after exposure to each individual compound, in the same amount as in the mixture. As shown in Fig 3, each of the components of the SVM significantly reduced the viability of J2s relative to that of unexposed J2s. However, 4-heptanone elicited the greatest reduction in viability: 90.8 ± 4.6% after exposure, which was similar to the effect elicited by the SVM (Fig 2). Assuming that the evaporation of each compound alone is similar to that obtained in combination with the other VOCs in the SVM, these results suggest that the decrease in *M*. *javanica* J2 viability after exposure to the SVM was mainly due to the presence of 4-heptanone. However, in light of our previous findings that the other components of the SVM played a role in control applications other than affecting *M*. *javanica* J2 nematodes namely against plant pathogenic fungi [48] we continued the experiments with the SVM and not with 4-heptanone only.

### 4-Heptanone nematicidal activity

**Fig 4:**
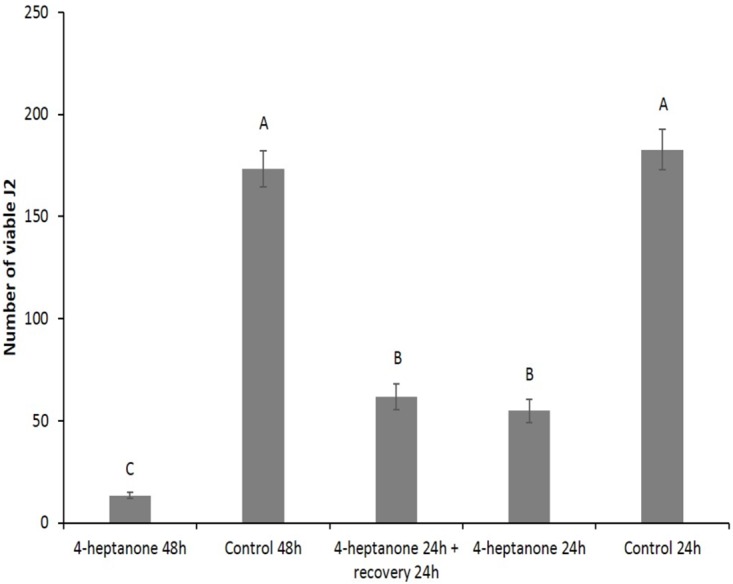
Nematicidal effect of the chemical compound 4-heptanone on *M*. *javanica* J2 viability. In each treatment the nematodes were incubated for 24 or 48 h in the presence or absence of 4-heptanone (0.4 mL, 2.86 mmole), before viable J2s were separated using 30 μm sieves. The numbers on the y-axis represent the numbers (mean ± SE) of viable J2s counted following the incubation. There were 8–10 repetitions, each using 300 J2s. In the treatment designated as "4-heptanone 24 h + recovery 24 h" the nematodes were exposed to the compound for 24 h and then removed to a new container with no VOCs for recovery. The results were subjected to analysis of variance followed by the Tukey-Kramer multiple comparison test; different letters above the bars indicate a significant difference between samples at *P ≤* 0.05. The experiment was independently repeated three times, each time with similar results.

In light of the finding that the most active component in the artificial mixture was 4-heptanone (Fig 3), we examined whether this compound behaved as a nematicide or was only nematostatic. To this end, we exposed J2s to 4-heptanone for 24 h and then allowed them to recover for 24 h, following removal of the compound. As a control, J2s were incubated for 24 or 48 h in the presence or absence of 4-heptanone. As shown in Fig 4, the number of viable J2s after a 24-h exposure to 4-heptanone did not differ significantly from the number of viable J2s present after a 24-h exposure followed by a 24-h recovery period in a new container with no VOCs, which implies that there was no recovery of the J2s following exposure to 4-heptanone. This result suggests that 4-heptanone exhibited a nematicidal effect on *M*. *javanica* J2s. Furthermore, as expected, exposure of J2s to 4-heptanone for 24 or 48 h resulted in significant reductions in the number of viable J2s relative to unexposed ones; and, moreover, this effect was significantly more pronounced after 48 h of exposure, which indicates the importance of the incubation period.

**Fig 5:**
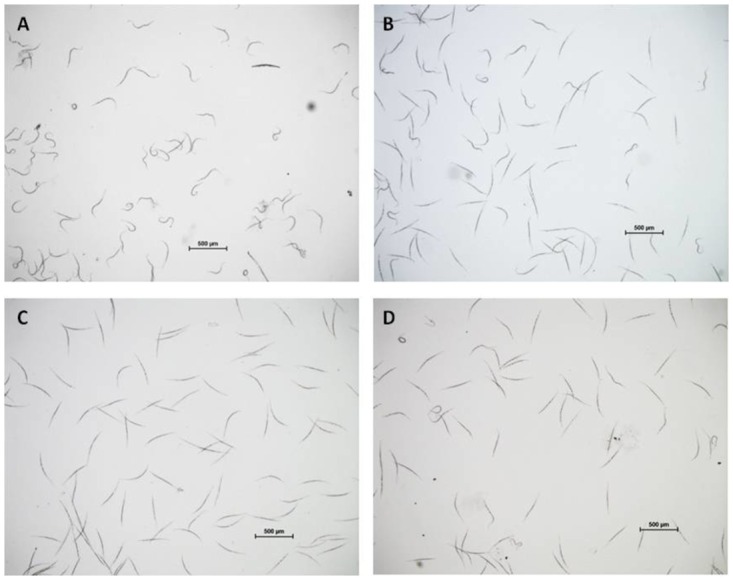
Phenotypes of Meloidogyne javanica J2 following exposure to D. cf. concentrica culture, SVM, and 4-heptanone. The nematodes were exposed to each compound for 48 h at 25°C in the dark, before visualization. A. Control—untreated nematodes. B. Nematodes exposed to three 50-mm-diameter culture plates, each with 5 mL of growth medium, on which *D*. *cf*. *concentrica* had been pre-grown for 4 days. C. Nematodes exposed to SVM preloaded onto 125 mg of perlite particles at 0.25 mL/L (V/V) [3-methyl-1-butanol (0.05 mL, 0.46 mmole), (±)-2-methyl-1-butanol (0.05 mL, 0.46 mmole), 4-heptanone (0.1 mL, 0.72 mmole), and isoamyl acetate (0.05 mL, 0.34 mmole)]. D. Nematodes exposed to 4-heptanone (0.05 mL, 0.36 mmole) preloaded onto 25 mg of perlite particles. The bar represents 500 μm.

Visual examination demonstrated that most of the J2s were mobile and retained the sinusoidal characteristic or curved shape typical for viable nematodes (Fig 5A). In contrast, after exposure to *D*. *cf*. *concentrica* culture plates, a clear reduction in the number of viable nematodes could be observed, as manifested in the higher proportion of nematodes with straight body shapes (Fig 5B). Moreover, after exposure to either the mixture or 4-heptanone (Fig 5C and 5D), most of the nematodes exhibited the typical straight body shape with hardly any curvature, implying reduction in viability. These observations are in accordance with the sieve test in which nematode movement and mobility through the sieve reflected the viability of the treated nematodes (Figs 1–4).

### VOCs effects on eggs

**Fig 6:**
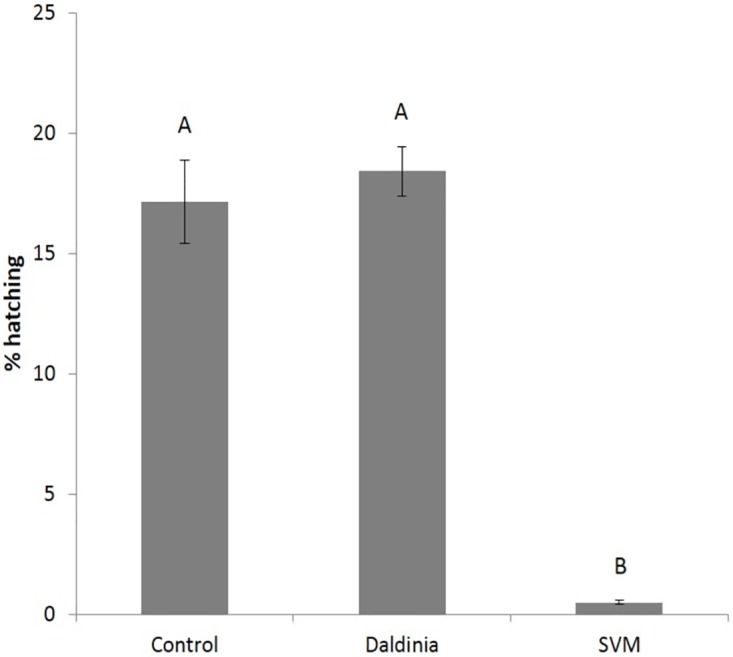
Effects of *D*. *cf*. *concentrica* volatiles and of the SVM on *M*. *javanica* eggs. The eggs were exposed for 48 h either to *D*. *cf*. *concentrica* grown in three 50-mm-diameter culture plates, each loaded with 5 mL of growth medium or to 0.25 mL/L (V/V) of the SVM [3-methyl-1-butanol (0.05 mL, 0.46 mmole), (±)-2-methyl-1-butanol (0.05 mL, 0.46 mmole), 4-heptanone (0.1 mL, 0.72 mmole), and isoamyl acetate (0.05 mL, 0.34 mmole)] preloaded onto 125 mg of perlite particles. Each treatment used 10 repetitions each using 800 eggs. The numbers on the y-axis represent the means (± SE) of percentage of egg hatching. The results were subjected to analysis of variance followed by the Tukey-Kramer multiple comparison test; different letters above the bars indicate a significant difference between samples at *P ≤* 0.05. The experiment was independently repeated three times, each time with similar results.

In light of the findings that both the fungus and the SVM affected the viability of J2s (Fig 2), we examined the susceptibility of *M*. *javanica* eggs in relation to hatching. As shown in Fig 6, the percentage of hatching of *M*. *javanica* eggs, which were exposed to *D*. *cf*. *concentrica* was similar to that of eggs under control, unexposed conditions. However, exposure to the SVM significantly reduced the percentage of *M*. *javanica* eggs hatching by 87.4 ± 8.7%, suggesting that the nematicidal activity of the SVM towards *M*. *javanica* eggs was stronger than that of *D*. *cf*. *concentrica* culture.

### Effect in nematode-infested soil

**Fig 7:**
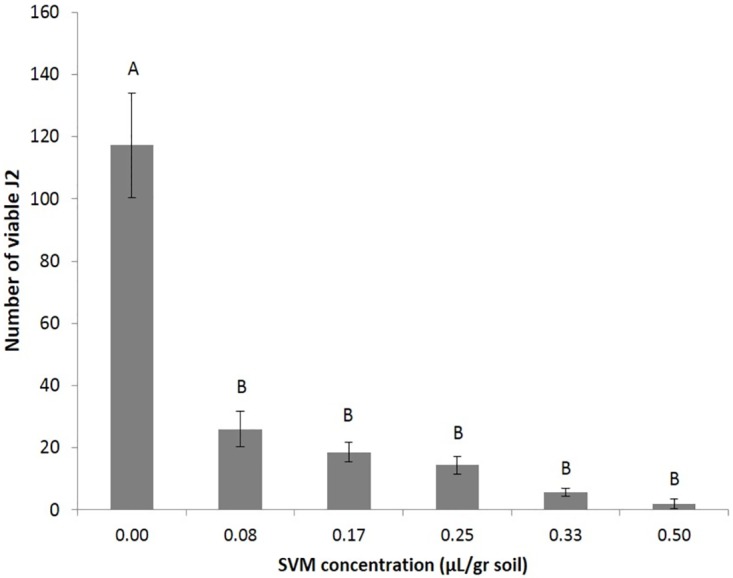
Activity of the SVM in loamy soil. A 0.4 mL stock of SVM [3-methyl-1-butanol (0.08 mL, 0.73 mmole), (±)-2-methyl-1-butanol (0.08 mL, 0.74 mmole), 4-heptanone (0.16 mL, 1.14 mmole), and isoamyl acetate (0.08 mL, 0.54 mmole)] was aliquoted, loaded on perlite particles, and mixed with 60 g of soil in a sealed 50-mL cup, before the addition of 500 J2s. The cups (five repetitions) were sealed and incubated for 48 h, after which viable J2s were determined according to the Baermann funnel method. The numbers on the y-axis represent the means (± SE) of viable J2s extracted from the soil. The results were subjected to analysis of variance followed by the Tukey-Kramer multiple comparison test; different letters above the bars indicate a significant difference between samples at *P* ≤ 0.05. The experiment was independently repeated twice, each time with similar results.

In order to examine the ability of the SVM to prevent parasitism development, we first evaluated its use to reduce J2s viability in soil. We mixed the soil with perlite particles preloaded with increasing volumes of the mixture, and then added *M*. *javanica* J2s to inoculate the treated soil. As shown in Fig 7, the SVM, at a concentration in soil as low as 0.08 μL/gr soil [3-methyl-1-butanol (0.001 mL, 0.009 mmole), (±)-2-methyl-1-butanol (0.001mL, 0.009 mmole), 4-heptanone (0.002 mL, 0.014 mmole), and isoamyl acetate (0.001 mL, 0.007 mmole)], significantly reduced larval viability. This result suggests that the mixture was effective against the nematodes in soil.

**Fig 8:**
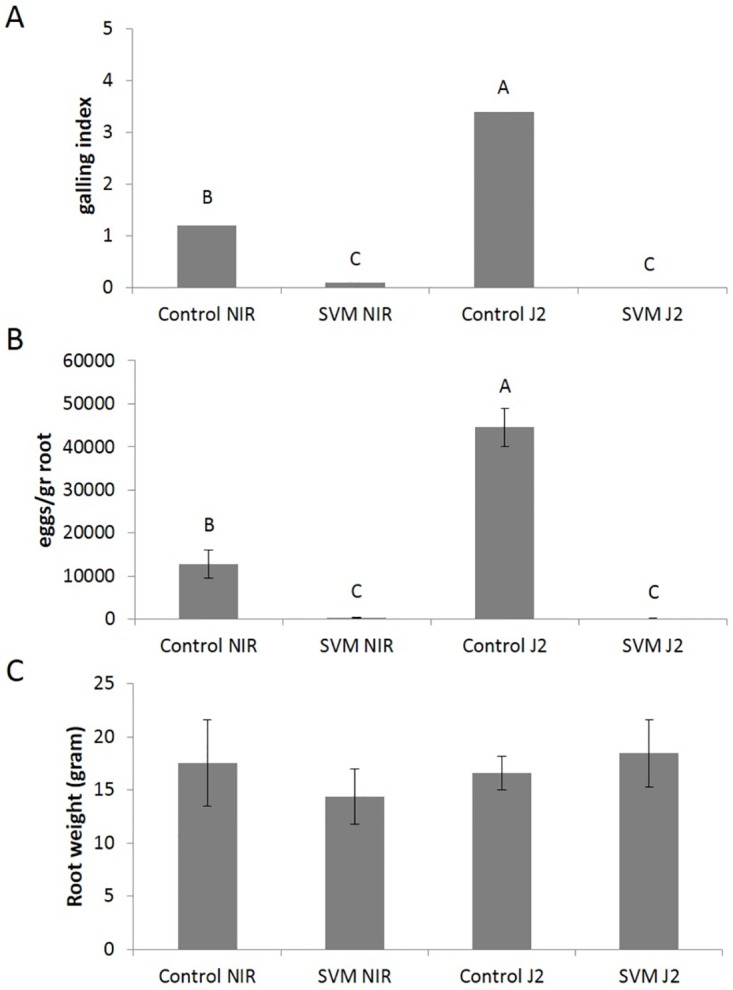
Effects of the SVM in greenhouse experiments. Susceptible tomato seedlings were planted in inoculated soil, with or without pretreatment with the synthetic volatile mixture (SVM). Four treatments were designated: Control NIR—soil inoculated with nematode-infected roots (NIR) infested by *M*. *javanica* in amounts equivalent to 4,000 eggs in root suspension in each pot; SVM NIR—soil inoculated with nematode-infected roots infested by *M*. *javanica* in amounts equivalent to 4,000 eggs in root suspension in each pot and supplemented with the synthetic volatile mixture; Control J2 –soil inoculated by direct insertion of 4,000 *M*. *javanica* J2s into each pot; and SVM J2 –soil inoculated by direct insertion of 4,000 *M*. *javanica* J2s and supplemented with the synthetic volatile mixture. (A) Galling index (mean of five repetitions). The results were subjected to nonparametric comparison using Wilcoxon method; different letters above the bars indicate a significant difference between samples significant at *P ≤* 0.05. (B) Mean numbers (± SE) of *M*. *javanica* eggs per gram of root. The results were subjected to analysis of variance followed by the Tukey-Kramer multiple comparison test; different letters above the bars indicate a significant difference between samples at *P ≤* 0.05. (C) Mean weights (± SE) of tomatoes roots. The results were subjected to analysis of variance. No significant differences (*P* > 0.01) between treatments in root weight were found. The experiment was independently repeated twice, each time with similar results.

Next, we inoculated susceptible tomato seedlings with the nematodes, in the presence or absence of the SVM, and after 7–8 weeks we evaluated disease occurrence as indicated by galling index, weighed the roots, and calculated the number of *M*. *javanica* eggs per gram of root. As shown in Fig 8, inoculated tomato plants grown in treated soil that was pre-exposed to the SVM exhibited significantly lower galling indexes and numbers of nematode eggs per gram of root than those grown in untreated soil, whereas there was no significant difference between the root weights of exposed and unexposed plants. Taken together, our results suggest that the SVM, based on four *D*. *cf*. *concentrica* volatiles, might form the basis of a new strategy to manage RKN.

## Discussion

### *D*. *cf*. *concentrica* exhibited activity against *M*. *javanica*

Accumulating evidence indicates the potential for using fungi as biological control agents against plant-parasitic nematodes [52–55]. Although examples of endophytic fungi with nematicidal effects were presented [41, 42], their modes of action are still obscure [54]. We demonstrated that the endophytic fungus *D*. *cf*. *concentrica* significantly affected J2s viability of the obligate parasite *M*. *javanica*. Interestingly, although we isolated *D*. *cf*. *concentrica* from an olive tree limb [48], i.e., far from the nematode's natural environment, it affected *M*. *javanica* viability. There are several possible explanations: A) the fungal VOCs might affect the free living or mycophagous nematodes in decaying wood, as competing with or feeding on the fungus, via an unknown mechanism; B) the fungal VOCs indirectly protect the tree from nematodes by increasing its resistance; or C) the fungal VOCs, which exhibit antifungal activity against various plant pathogenic fungi, may assist *D*. *cf*. *concentrica* to survive inside the plant tissue [48], and may also happen, by a side effect on an evolutionary-conserved pathway, to affect nematodes. Further studies are needed to examine these possibilities.

### Evaluating the advantages of using SVM

Our results indicate that the SVM provided better control of *M*. *javanica–*both its J2s (Fig 2) and its eggs (Fig 6)–than *D*. *cf*. *concentrica* culture plates. Similarly, a mixture of the natural pesticidal compounds of *M*. *albus* exhibited better control against the RKN *Meloidogyne incognita* than treatment with the *M*. *albus* culture [42]. These higher activities may reflect the presence of higher concentrations of the chemical compounds in the SVM than in the fungal natural emissions, and/or absence of other volatiles that are present in the fungus and that could interfere with its nematicidal activity. Furthermore, because fungal production of specialized metabolites is controlled by the circadian rhythm, accumulation of these compounds might not be continuous through all developmental stages [56].

### SVM competence, uniqueness, and novelty

The toxicity of our SVM has not been tested yet, however, all the four components are used in the food industry **(**http://www.sigmaaldrich.com/industries/flavors-and-fragrances.html), and therefore their use in nematode control should be permissible. Although each of the four components significantly reduced J2 viability, only 4-heptanone affected the nematodes similarly to the whole mixture (Fig 3), and exhibited a nematicidal activity (Fig 4). To the best of our knowledge, the present paper is the first report of a nematicidal effect of 4-heptanone on a plant parasitic nematode. The mechanism by which 4-heptanone affects J2s is still obscure; however, preliminary results demonstrated that 4-heptanone affected the viability of *Caenorhabditis elegans* also (data not shown). Assuming that the behavior of *M*. *javanica* is similar to that of *C*. *elegans*, this finding indicates that this model nematode could be used to elucidate the mechanism of action.

Among the four chemical components of our SVM, three are already correlated with plant parasitic nematode activity: 2-methyl-1-butanol [43] and 3-methyl-1-butanol [42, 43] were ingredients of SVM that exhibited nematicidal effects against *M*. *javanica* [43] and *M*. *incognita* [42]. Although the effect of each individual component was not determined in those studies [42, 43], our present results confirm their nematicidical activity (Fig 3). However, the effect of the third component, isoamyl acetate, depended on the nematode: although it was a potent attractant for *C*. *elegans* [57], it was neutral toward the dauer juvenile stage of *Heterorhabditis bacteriophora* [58] and was nematicidal to *M*. *javanica* (Fig 3). It should be noted that in contrast to our present study, which focused on *D*. *cf*. *concentrica* VOCs, a previous study identified two naphthalene derivatives from *D*. *concentrica* culture that showed nematicidal activities [59].

Application of our SVM to *M*. *javanica*-inoculated soil in the presence of nematode-susceptible tomato seedlings nearly abolished gall formation and significantly decreased the number of nematode eggs per gram of root (Fig 8). This result is consistent with our findings that the SVM was active against *M*. *javanica* eggs (Fig 6) and in soil (Fig 7). Successful greenhouse experiments with volatiles were previously reported: VOCs produced by *Fusarium oxysporum* isolated from the soil rhizosphere of coffee plants decreased *M*. *incognita* infectivity on tomato [44]; the endophytic fungus *M*. *albus* significantly reduced levels of the nematodes *Meloidogyne chitwoodi*, *Meloidogyne hapla*, *Paratrichodorus allius* and *Pratylenchus penetrans* in host roots and rhizosphere soil [41]. Furthermore, a SVM based on *M*. *albus* VOCs effectively reduced the number of *M*. *incognita* galls per tomato plant [42]. In light of the facts that the activity of the SVM was stronger than the activity of the living organism (Fig 2, [42]), and that the use of a live microorganism as a biocontrol agent must take into account the limitations of life-supporting conditions to be active and effective, we propose the application of our SVM as the basis of an alternative safe approach for controlling the RKN *M*. *javanica*.
